# Health trajectories after age 60: the role of individual behaviours and social contexts

**DOI:** 10.1093/geroni/igab046.2327

**Published:** 2021-12-17

**Authors:** Amaia Calderón-Larrañaga, Xiaonan Hu, Miriam Haaksma, Debora Rizzuto, Laura Fratiglioni, Davide Vetrano

**Affiliations:** 1 Karolinska Institutet, Solna, Stockholms Lan, Sweden; 2 Leiden University Medical Center, Leiden, Zuid-Holland, Netherlands; 3 Karolinska Institutet, 17175, Stockholms Lan, Sweden

## Abstract

This study aimed to detect different health trajectories after age 60, and to explore to what extent individual and social factors may contribute to healthier ageing. Twelve-year health trajectories were identified in subjects from the Swedish National Study on Aging and Care-Kungsholmen (N=3108), integrating five indicators related to diseases, physical and cognitive function, and disability by means of nominal response models. Growth mixture models were applied to explore health trajectories in terms of rate and pattern of change. Baseline information about health-related behaviours and social context was collected through standardized questionnaires. The strength of the associations was estimated using logistic regression, and their impact through population attributable fractions (PAF). Three trajectories were identified grouping 78%, 18%, and 4% of people with respectively increasing rates of health decline Compared to the best trajectory, subjects in the middle and worst trajectories became functionally dependent 12.0 (95%CI:11.4-12.6) and 12.1 (95%CI:11.5-12.7) years earlier, respectively. Insufficient physical activity (OR:3.38, 95%CI:2.58-4.42), financial strain (OR:2.76, 95%CI:1.77-4.30), <12 years education (OR:1.53, 95%CI:1.14-2.04), low social connections (OR:1.45, 95%CI:1.09-1.94), low social participation (OR:1.39, 95%CI:1.06-1.83) and a body mass index ≥25 (OR:1.34, 95%CI:1.03-1.75) were associated with belonging to the middle/worst trajectories. The highest PAFs were observed for insufficient physical activity (27.1%), low education (19.3%) and low social participation (15.9%); a total PAF of 66.1% was obtained when considering all significant exposures together. Complementarily considering life-long factors belonging to the socioeconomic, psychosocial, and behavioural dimensions should be central to any strategy aimed at fostering health in older age.

